# Cross‐sector pre‐registration trainee pharmacist placements in general practice across England: A qualitative study exploring the views of pre‐registration trainees and education supervisors

**DOI:** 10.1111/hsc.13783

**Published:** 2022-03-15

**Authors:** Ali M. K. Hindi, Sarah C. Willis, Ellen I. Schafheutle

**Affiliations:** ^1^ Centre for Pharmacy Workforce Studies Division of Pharmacy and Optometry The University of Manchester Manchester UK; ^2^ School of Health Sciences The University of Manchester Manchester UK; ^3^ Faculty of Biology, Medicine and Health The University of Manchester Manchester UK

**Keywords:** clinical supervision, experiential learning, foundation training, general practice, placements, pre‐registration pharmacy training, primary care

## Abstract

The Pharmacy Integration Fund commissioned 95 cross‐sector pre‐registration trainee pharmacist placements across England, which incorporated trainees spending 3–6 months in general practice (GP), whilst employed in hospital or community pharmacy. Delivery models varied (blocks or split weeks/days); trainees had pharmacist tutors at the employing/base (hospital/community pharmacy) organisation and in GP. This study aimed to evaluate implementation of cross‐sector pre‐registration placements, and to identify barriers and enablers of a “successful” placement that achieved its intended outcomes. A qualitative study was undertaken, using semi‐structured interviews with triads/dyads of trainee and pharmacist tutors at base and/or GP site. Interviews explored trainees’ and tutors’ GP placement experiences, and the contribution of GP placements to achieving intended learning outcomes. Data were thematically analysed. Thirty‐four interviews (14 trainees, 11 base tutors, 9 GP tutors) were completed in 11 study sites (5 GP/hospital; 6 GP/community pharmacy). GP placements were perceived as valuable and producing well‐rounded pre‐registration trainees with a good understanding of two settings. Key benefits of GP placements were trainees’ ability to work within multidisciplinary teams, and improved clinical and consultation skills. Contingency planning/flexibility was important when setting up cross‐sector placements. GP tutor supervision which supported a gradual transition from shadowing to more independent clinical practice with feedback was perceived as valuable. Good collaboration between tutors at the base and GP site ensured joined‐up learning across settings. All participants considered 13 weeks in GP an appropriate minimum duration; community trainees preferred longer duration (26 weeks) for more opportunities for clinical and consultation skills learning. Base and GP tutors would welcome clarity on which pre‐registration competencies should be achieved in GP placements, which would also aid quality and consistency across providers. Findings from this study identified key attributes of a successful pre‐registration cross‐sector training experience. These findings can inform policy reforms including changes to initial education and training of pharmacists.

**What is known about this topic?**

- Pharmacists traditionally spent their pre‐registration year in community or hospital, with variation between settings.
- Increasingly, pharmacists are employed in patient‐facing primary care settings, and their trainings need to adequately prepare them for these patient‐facing roles.

**What this paper adds**

- Cross‐sector pre‐registration placements in general practice (GP) improve trainee pharmacists’ understanding of patient pathways and holistic patient care.
- General practice placements particularly support trainee pharmacists’ development of consultation and clinical assessment skills and multidisciplinary team working.
- Key considerations when implementing cross‐sector GP placements include: good operational planning; collaborative supervision; well‐supervised workplace learning in a supportive GP environment with appropriate opportunities for trainees to learn and harness skills.

## 1 INTRODUCTION

In recent years, pharmacists’ roles in England have changed (NHS England, 2014, 2019), with increasing numbers working in a range of primary care settings, that is, general practice (GP – family medicine), urgent care, and care homes. The vision for a fit‐for‐purpose pharmacy workforce sees pharmacists able to work across integrated care pathways, providing patient‐centred care and medicine optimisation. Similar movements to integrate pharmacists within primary care teams can be seen internationally, such as in Canada (Raiche et al., 2020; Samir Abdin et al., 2020), the United States (Jacobi, 2016), Australia (Moles & Stehlik, 2015), and Malaysia (Saw et al., 2017). Reported benefits of pharmacists working with GPs include controlling prescribing expenditure, detecting and resolving drug‐related problems, and making clinical interventions to patients’ medicines (Khaira et al., 2020; Mann et al., 2018).

The NHS Long Term Plan (2019) sets out proposals to significantly grow the number of pharmacists in primary care (NHS England, 2019), and to ensure that as independent prescribers they become a central part of multidisciplinary primary care teams. There are currently more than 1000 full‐time equivalent pharmacists working in GPs as well as urgent care settings and care homes, with funding available through the NHS England Pharmacy Integration Fund and the GP five‐year contract framework (NHS England, 2021).

Delivering the NHS Long Term Plan will also require reform to initial education and training for pharmacists, who in Great Britain mainly undertake 4 years of university‐based education followed by 12 months of work‐based pre‐registration training^1^ where they are supervised by a pharmacist tutor (Sosabowski & Gard, 2008).

Unlike medicine or nursing, undergraduate pharmacy education is funded as a science degree and incorporates limited experiential learning, with the pre‐registration year currently contributing the main patient‐facing experience prior to registration. Until 2021, pre‐registration trainees have had to meet 76 General Pharmaceutical Council (GPhC) set performance standards, against which their tutor signs them off during formal meetings after 13, 26, and 39 weeks (General Pharmaceutical Council, 2020). Following a final tutor sign‐off, trainees need to pass the GPhC registration assessment in order to apply for pharmacist registration.

Limited undergraduate experiential learning and the traditional set‐up of pre‐registration training taking place in a single sector, usually hospital or community pharmacy, create the challenge of achieving a sustainable pharmacy workforce that has the knowledge, skills, and understanding to work in primary care and across the wider integrated care system (NHS England, 2016; NHS Health Education England., 2016). Pre‐registration placements in GP provide a possible solution to this challenge.

### 1.1 Pre‐registration trainee pharmacists in general practice project (2019–current)

In 2019, the Pharmacy Integration Fund commissioned the Pre‐registration Pharmacists in General Practice Project, where 95 trainee pharmacists were employed in a base sector (community or hospital pharmacy) but spent between 13 and 26 weeks in GP throughout England. These GP placements were managed by Health Education England (HEE), the NHS statutory body responsible for the education and training of the health workforce. HEE appointed a national lead and regional facilitators to advise and support trainees, tutors, employers, and host sites in the development and delivery of GP placements. They also developed resources for base and particularly GP host sites including GP placement objectives, expected outcomes, and a framework outlining how to meet both the GPhC performance standards and HEE recommended outcomes (Appendix S1).

The structure of cross‐sector placements varied, encompassing one or more blocks, and weeks or days split between the base sector and GP setting. Trainees had a pre‐registration pharmacist tutor at the base sector, who retained overall responsibility for the trainee throughout the year, and a second pharmacist tutor working in GP placements who understood the scope of practice of the still emerging role of a primary care pharmacist (NHS Health Education England., 2020).

Whilst in GP, trainees completed a reflective e‐portfolio to demonstrate competence against the GPhC performance standards. All trainees and tutors had access to this e‐portfolio, which included a number of formative assessment tools (Appendix S2).

The **overall aim of this study** was to evaluate implementation of cross‐sector GP/community and GP/hospital pre‐registration placements in England, and to identify barriers and enablers of a training placement that achieved its intended outcomes for learners – conceptualised here as a ‘successful training placement’.

The purpose of this paper is to use our evaluation findings to shed light on how to best implement cross‐sector placements.

## 2 METHODS

### 2.1 Study design and sampling

A qualitative study design was used, with study sites in England purposively selected on the basis of key situational variables (Gray, 2019):

- Pharmacy base: community and hospital
- Number of pre‐registration trainee pharmacists in base doing GP rotation
- Length of GP placement: 13 weeks versus 26 weeks
- Organisation of GP placement: block versus split week/day
- Regions within England

At each study site, semi‐structured telephone interviews were conducted with trainees, pharmacy base tutor, and/or GP pharmacist tutor, using a dyad/triad approach. A dyad involved at least one trainee and one of their tutors being interviewed. A triad involved at least one trainee and both their base supervisor and GP tutors. Study sites had to have a trainee and tutor participate to be included in the study.

### 2.2 Recruitment

The HEE national project lead provided the research team with 78 training sites and characteristics for purposive sampling. The research team initially selected 8–12 study sites using a sampling matrix based on key situational variables described above and emailed invitation letters and participant information sheets (PIS), with a request to contact the research team. These assured participants of confidentiality and that they could withdraw from the study without impact on their training. If sites from the initial sampling matrix didn't wish to participate, they were replaced by other sites with similar characteristics.

### 2.3 Data collection

Telephone interviews were conducted with trainees and tutors at seven study sites between January and March 2020; interviews were paused due to the emerging COVID‐19 pandemic, and resumed in June to July 2020. All participants provided written or verbal consent before the interview commenced. Interview schedules were informed by existing research (Jee et al., 2016, 2017, 2019; Jones et al., 2019; Schafheutle et al., 2017), an earlier pilot evaluation (Gray, 2019), and the HEE‐GP pre‐registration handbook (NHS Health Education England., 2020). Schedules were revised following discussions with the HEE national lead, with questions tailored to understand the contribution of GP placements to the achievement of pre‐registration learning outcomes, and an opportunity at the end to reflect on their overall GP placement experience (Appendix S3).

This study received ethics approval by The University of Manchester Research Ethics Committee (Ref no. 2020‐7914‐16,794) and NHS Health Research Authority (Ref no. NHS001659).

### 2.4 Data analysis

All interviews were audio‐recorded and transcribed verbatim. Interview transcripts were analysed by the first author, aided by NVivo 11 (QSR International Pty Ltd, 2015), using inductive data‐driven coding followed by thematic analysis to provide rich detailed descriptions (Braun & Clarke, 2006), focussing on the exploration of inter‐ and intra‐group themes. Analysis and themes were discussed with the co‐authors in regular meetings throughout analysis. Interpretation of findings were then checked with the programme national lead and relevant contacts from NHSE PhIF.

## 3 RESULTS

### 3.1 Site characteristics

The characteristics of placement sites involved in this study are provided in Table 1. Of 33 placement sites approached, 11 participated as a dyad/triad (i.e. trainee and at least one of their tutors) [Table 2]. Reasons for non‐participation are provided in the report (Hindi et al., 2021). Thirty‐four interviews were completed (14 trainees – 6 female, 8 male; 11 base tutors – 4 female, 8 male; 9 GP tutors – 4 female, 5 male). In one placement site, the superintendent (pharmacist with overall responsibility across a pharmacy chain) was interviewed instead of the base tutor.

**TABLE 1:** Characteristics of placement sites involved in study (*N* = 11)

Characteristics of pairings	Total coverage in sampling frame
Pharmacy base	Community pharmacy (*n* = 6)^a^ Hospital (*n* = 5)^b^
Organisation of GP placement	Single block (*n* = 3) Multiple blocks (*n* = 2) Split week (*n* = 5) Split day (*n* = 1)
Region	North (*n* = 3) South (*n* = 2) London and South East (*n* = 2) Midlands and East (*n* = 4)
Number of trainees in GP setting	Multiple trainees (*n* = 3) Single trainee (*n* = 8)
Length of GP placement	13 weeks (*n* = 4) <13 weeks (*n* = 1) 26 weeks = (*n* = 6)

^a^ **Community pharmacy:** independent (*n* = 1), small = 6–25 chains (*n* = 1), medium = 25–200 chains (*n* = 2), large = 200 + chains (*n* = 2).

^b^ **Hospitals:** university (*n* = 3), district general (*n* = 1), specialist (*n* = 1).

**TABLE 2:** Participants interviewed in the study (*N* = 34)

Study sites	Organisation	Duration	Participants interviewed
Site 1 (hospital/GP)	One block	8 weeks^a^	Trainee, Base tutor, GP tutor
Site 2 (hospital/GP)	Split weeks	26 weeks	Trainee 1, Trainee 2, Base tutor
Site 3 (hospital/GP)	One block	13 weeks	Trainee 1, Trainee 2, Base tutor, GP tutor
Site 4 (hospital/GP)	Multiple blocks	13 weeks	Trainee, Base tutor, GP tutor
Site 5 (hospital/GP)	Single block	13 weeks	Trainee, Base tutor, GP tutor
Site 6 (community/GP)	Split day	26 weeks	Trainee, Base tutor, GP tutor
Site 7 (community/GP)	Multiple blocks	26 weeks	Trainee, Base tutor, GP tutor
Site 8 (community/GP)	Split weeks	26 weeks	Trainee 1, Trainee 2, Base tutor, GP tutor
Site 9 (community/GP)	Split weeks	26 weeks	Trainee, Base tutor
Site 10 (community/GP)	Split weeks	13 weeks	Trainee, Base tutor, GP tutor
Site 11 (community/GP)	Split weeks	26 weeks	Trainee, Base tutor, GP tutor

^a^ Original placement provider dropped out prior to the start of the programme. Replacement site only offered an 8‐week GP placement.

### 3.2 Overview of GP placement model

**FIGURE 1:**
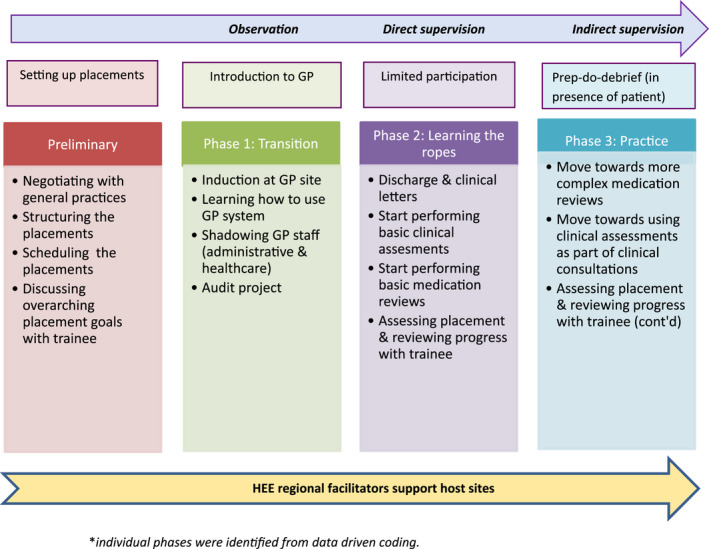
Overview of GP placement model^*^ ^*^Individual phases were identified from data driven coding

Trainees viewed the more flexible structure of GP placements with overarching goals, which was more learner‐centred and tailored to their needs than their base setting as important. During thematic analysis, findings were materialised into a model for implementation of cross‐sector placements in GP involving a number of key phases (Figure 1), which are described next.

### 3.3 Preliminary phase

This phase covers setting up/planning cross‐sector placements, and what needs to be in place prior to trainee arriving.

#### 3.3.1 Setting up cross‐sector placements

Setting up “successful” training placements required negotiation with GP sites to take on trainees, which was more straightforward when building on already established relationships. Because base sector and GP sites had to register with HEE a long time in advance, contingency planning/flexibility was needed to allow for changes in staffing and circumstances in base and GP site.

#### 3.3.2 Preparing for GP placements

An orientation event provided an important opportunity for trainee and base tutors to meet and discuss expectations, outcomes, and the placement structure. Many base tutors arranged for trainees to meet their GP tutors before the placement started, some arranged for the trainee to visit the GP site.

#### 3.3.3 GP placement models

Employer (base) and host GP practice sites needed to negotiate and agree on how to structure GP placements. Trainees and tutors highlighted advantages and disadvantages of different placement structures. Hospital tutors and trainees in single block placements believed that this structure enabled trainees to fully integrate in GP by spending uninterrupted time there. They also viewed preferred block placements as fitting well with a hospital's rotation structure. GP tutors perceived a block made it easier to incorporate a trainee into routine practice.

“I think it’s better that it’s a block because I think it gives better continuity, it allows the pre‐regs to settle in because I think it is difficult for our pre‐regs rotating through these different areas and having to learn about new systems, new environments, new staff that they’re working with. I feel like they need to settle into the new rotation and set objectives that are consistent”. (Site 5, hospital, base tutor – single block)

Both base and GP tutors in the two study sites perceived their multiple block placements enabled spiralling of learning (i.e. spread out over time rather than being concentrated in shorter periods). The main disadvantage of block placements was that it required trainees to relearn or refresh their understanding upon returning to base sector.

Most community pharmacy/GP pairings used split week placements, which were viewed as helping trainees to develop in both sectors simultaneously throughout the year, and as enhancing cross‐sector communication between community pharmacy and GP.

“I really enjoy the split weeks. It’s really nice to work on patient cases in both GP and in the community pharmacy […] And just building the relationship with both the colleagues in the community pharmacy and in the GP practice …. I think it's helped the community pharmacy’s communication with the GP practice”. (Site 9, community pharmacy, trainee – split weeks)

Some trainees identified that split week placements meant not always being able to see through the resolution of problems, which was more pronounced in split day placements (morning GP, afternoon community pharmacy).

“To a certain extent, it’s good, but I mean, there are opportunities or certain incidents where I miss certain components of the day to day activity of either or both the GP or the community. So, for example, because I leave early at the GP, I don’t see like the med reviews that happen towards the afternoon. Or if I’m in the pharmacy, I don’t actually do dispensing of the methadone or something like that, for patients who come in the morning. So I kind of sometimes miss aspects of both, but I have like snippets”. (Site 6, community pharmacy, trainee – split days)

#### 3.3.4 Duration and timing of GP placement

GP practice and base site agreed their preferred placement duration and its timing. Most trainees and base tutors in block placements preferred trainees to spend their initial 3 months in the base sector (until the 13‐week appraisal), so that trainees became accustomed to their base site, build confidence, and complete some of their hospital accreditations and logs (where relevant).

All trainees and tutors in the hospital/GP pairings agreed that 13 weeks was an appropriate (minimum) duration offering with sufficient opportunities to undertake a range of activities and learn new skills. Trainees, hospital base, and GP tutors considered that 26 weeks in GP would make fitting in all hospital activities challenging.

“I think less than nine months there, three months with us probably wouldn't be sufficient to cover everything you need to cover in hospital. All that you would do if you stayed longer is just develop further… So you wouldn't necessarily do any more in terms of what you do, it would just be more complex and possibly more independent if you stayed longer.” (Site 3, hospital, GP tutor – single block)

In community pharmacy/GP pairings, all trainees and base (except one) and GP tutors considered 26 weeks in each sector optimal.

“I'd probably say it's perfect the way it is 26 weeks because as much as there is to do in GP, there is always a lot to do in community pharmacy as well. So I think if you're in one place more than the other, then you're kind of missing out in either place”. (Site 8, community pharmacy, trainee 2 – split weeks)

### 3.4 Collaboration between base and GP sites

Once GP placements were underway, base and GP tutors emphasised the importance of good communication particularly at handover to ensure all processes/procedures were set up for the trainee. Base tutors highlighted the importance of keeping the trainee linked to the base sector, by making sure that trainees had access to regular learning sets and training days at the base during their GP placement.

### 3.5 Phase 1: Transition

This phase is about how trainees were introduced to the GP environment and factors which eased/supported trainees’ transition from base to GP sector.

#### 3.5.1 Commencing GP placements

Analysis showed the importance of GP sites to understand the pre‐registration trainee role, in terms of competence and scope of practice as non‐registered healthcare professionals. Trainees believed that GP staff were prepared for them at the practice to start their placement, but were commonly unclear about a trainee pharmacist's capabilities (i.e. skills and knowledge). This meant that clinical and non‐clinical staff had to spend time initially to better understand what trainees could be expected to do in GP.

“I don’t know if they knew what they wanted me to do…and I didn’t really fit anywhere, but as time went on, obviously they figured out what I can do, what I’m comfortable doing, what I’m not comfortable doing, and therefore obviously created a template around me, and that will feel like I’m contributing to the team”. (Site 6, community pharmacy, trainee – split day)

#### 3.5.2 Supporting trainees’ transition to GP sector

When first entering their GP setting, trainees needed to adapt to the new work environment, and building rapport with staff, with a range of factors easing transition and creating a supportive learning environment. A positive welcome and an effective induction covering policies, procedures, and mandatory training was vital. To begin with, trainees spent time shadowing non‐clinical staff in order to get to know the IT system, how to book patient appointments, scanning in clinic letter, and referring patients on, although trainees did not immediately recognise the value of shadowing:

“The feedback I got from the trainee was that she initially didn't understand why she needed to do those things, because it was an administrative task, it was something that a receptionist does. But after discussion she understood the purpose of doing those tasks is to get a wider understanding of how everything fits together in general practice, how things are triaged, how people end up in certain clinics and once that was explained to her she appreciated the task in hand a bit better. (Site 4, hospital, GP tutor – multiple blocks)

### 3.6 Phase 2: Learning the ropes

This relates to the phase in the supervision model which supports trainees’ gradual transitioning from shadowing to more independent clinical practice, by starting to perform activities.

#### 3.6.1 Activities undertaken by trainees and supervision to support work‐based learning

Following the induction/shadowing period, trainees performed a range of activities that gradually increased in complexity, progressing from technical and administrative tasks (e.g. medication queries, medication reconciliations) to clinical tasks (e.g. medication reviews, basic clinical assessments). Supervision also changed over time, depending on a trainee's confidence and competence and the nature of the activity.

With time, trainees became more capable of performing clerical tasks, audits, dealing with different kinds of medication queries on the telephone (e.g. queries about patient medications, repeat prescriptions), and reconciling medications for patients recently discharged from hospital. It took trainees time to undertake patient‐facing activities such as clinical assessments and medication reviews. GP tutors supported trainees to gradually take on an increasingly active role. In the beginning, trainees would observe their GP tutors undertake medication reviews. In preparation, tutors asked trainees to go through a patient's medicines, identify any problems, discuss changes, and potential discussion points with patient.

“We watched the pharmacist do medication reviews and then he’d kind of give us patients that were coming in and research into the problems they might be having; going through their medication list, picking out any kind of health thing we want to do. It was kind of doing what they’re doing but in the prep beforehand, obviously, because we weren’t experienced enough to do it ourselves”. (Site 2, hospital, trainee 2 – split weeks)

Following a period of observation and trainees developing their clinical skills (Table 3), supervision progressed to GP tutors selecting patients prescribed a single medication or who required a single chronic disease medication review and asking trainees to consult under supervision.

“I’ll pick out at least one or two patients from that list for them to actually do the review with me sitting in with them. So they’re starting to do the consultation skills, they might have to do a blood pressure check, they might have to do a peak flow”. (Site 3, hospital, GP tutor – single block)

**TABLE 3:** Clinical skills

Basic clinical skills
Weight Height BMI Heart rate Respiratory rate Temperature Blood pressure Oxygen saturation Urinalysis Capillary blood glucose Peak flow

### 3.7 Phase 3: Practice

The practice phase of the implementation model relates to when trainees undertake more complex medication reviews and clinical assessments, underpinned by a medical education supervision model.

#### 3.7.1 Pre‐brief to debrief (in presence of patient)

As trainees learned how to apply their clinical knowledge, they moved to provided face‐to‐face medication reviews more independently, with most GP tutors basing their approach on that used with undergraduate medical students:

“We’ve used the same structure as what we would do for the undergraduate medical students…. where he will see a patient and we’ll protect some time straight after, you know, for the supervisor which is myself. Then he’ll see the next patient and then there’ll be some protected time to debrief in front of the patient. So, we’ve used the same for the pre‐reg pharmacists and that seems to work really well because then he’s got confidence that if there’s something he’s unsure about, there’s going to be somebody, you know, there straightaway for him to handover to”. (Site 7, community pharmacy, GP tutor – multiple blocks)

Whilst trainees learned different consultation styles and refined their clinical skills through also observing nurses and GPs during clinics, trainees reported very limited engagement with trainees from other healthcare professions.

### 3.8 Feedback and assessment during GP placements

Trainees felt their GP tutors were very supportive and approachable, and reported having open and regular communication. GP tutors facilitated trainees’ learning and development by providing learning opportunities and formative feedback. This involved GP tutors discussing key learning objectives for activities; asking trainees thought‐provoking questions; and signposting to resources for self‐study.

Having a shared/joint approach between sites to supporting trainees to achieve intended learning outcomes was important. However, some GP and base tutors strongly believed that in future, GP placements needed to be underpinned by a framework for assessing trainees’ competence to undertake patient‐facing activities. Furthermore, base and GP tutors sought reassurance that they were providing the GP placement appropriately, particularly as this type of cross‐sector placement was still in its infancy.

“…there’s no competency framework for pre‐regs, so this is where we struggled a bit. But it’s a case of how many times do you get them to check a temperature or listen to a chest or do a peak flow before you can say that they’re competent to do it on their own, given the fact they did it for four to five years as part of the undergraduate degree as well.” (Site 7, community pharmacy, GP tutor – multiple blocks)

GP tutors at all of the study sites only discussed the formative assessment tools (Appendix S2) when prompted. It became clear that tutors either used these tools rarely or not at all.

### 3.9 Placement outcomes

When trainees and base and GP tutors were asked about the benefits and drawbacks of a GP placement, all thought that trainees could apply the knowledge gained at university in practice, and that their consultation and clinical skills significantly improved.

“With consultation skills, for pharmacists anyway I feel it’s something we don’t do enough of at university… you never really develop how to speak to a real person in front of you. So I think that’s an important part of what I try and do here is to develop those skills, because I think they’re the ones that we’re missing as pharmacists. And it is something that you have to develop your own way of consulting. So you can watch other people and see how they do it, but you need to develop your own way and your own confidence, and it’s nice to see that over the 13 weeks you see that starting to develop”. (Site 3, hospital, GP tutor – single block)

All participants agreed that experiencing two different sectors produced a well‐rounded trainee pharmacist who could work in both sectors. Cross‐sector working also enabled trainees to be flexible/adaptable, learn new skills quickly, and form new relationships with different members of the multidisciplinary team. Trainees in both types of pairings gained a better understanding of the patient pathway across different care settings, and they appreciated the importance of good communication between settings.

“I think it gives you a really good holistic view of healthcare, in that I think I’m now much more able to understand a patient’s journey from GP to hospital. But I think the bigger benefit of that actually is me understanding the importance of communication between the two sectors. […] In hospital you are told to make sure your discharge summaries are clear, but now I’ve actually seen the other end of it and had to fix those things”. (Site 1, hospital, trainee – single block)

“Now I can work in two different places quite seamlessly. I think you learn to be a bit more flexible in your working and adapt in that sense as to what you’re doing on a daily basis. I think as well as that it helps that you’ve seen the whole process of primary care really – well, almost anyway – to see how medications are prescribed, reauthorized, sent across to the pharmacy and then dispensed.”. (Site 7, community pharmacy, trainee – multiple blocks)

As placements progressed, trainees and GP tutors felt they became a valued member of the team who helped ease some of the GP workload:

“At first I did kind of feel like I was a weight or a burden to obviously the GP, because I had to be taught everything from the beginning, but as time went on, I do feel like I’m being invited more, and people are coming to me more and asking me, can you help with this, or can you help with that issue.”. (Site 6, community pharmacy, trainee – split day)

Most tutors felt that time commitment and procedure to run a cross‐sector placement was similar to single‐sector training. Trainees were supernumerary, so impact on day‐to‐day practice was minimal.

What created some difficulty was a lack of flexibility in delivery/organisation of hospital/GP placements, which meant that hospital/GP trainees were expected to complete the same logs, assessments, etc. as those undertaking single‐sector training:

“But I found that in hospital mainly I would be behind in a lot of things. So, for example, my dispensing competencies. Because I had GP in it the way that they structured my pre reg year they kind of cut certain rotations that normally for the other previous years would be two weeks, now mine is one week, or it would be four weeks, now mine is three weeks. But they’ve kind of kept the same expectations as if I was there the whole time. So because of that I found that I struggled in hospital because I have limited time to do something. (Site 4, hospital, trainee – multiple blocks)

Split community pharmacy/GP trainees and tutors were more concerned about trainees missing opportunities to learn the management side of community pharmacy (i.e. how to run a branch and manage people).

## 4 DISCUSSION

This study explored views and experiences of cross‐sector GP/community and GP/hospital pre‐registration pharmacy placements, with a view to make recommendations for how to design and deliver multi‐sector learning. The study used a qualitative triad (dyad) approach involving 11 study sites, whereby the trainee and their tutor(s) were interviewed.

Findings from this study have been applied to design a model (Figure 1) to inform policy makers in relation to implementation of cross‐sector pre‐registration trainee placements in GP. Key factors to consider when rolling out this type of placement more widely include: good operational planning of GP placements and appropriate induction; collaborative supervision grounded in effective communication and working relationship between base and GP tutors; learner‐centred and well‐supervised workplace learning in a supportive GP environment with appropriate opportunities for trainees to learn and harness skills; and clear integration of GP placements and intended learning outcomes/competencies across the whole training year.

Our findings indicate that GP placements should be progressive, increasing in complexity from shadowing and observation, onto simple tasks to application of consultation and clinical skills. This is consistent with medical supervision (Merritt et al., 2017), whereby learning should start with shadowing and observing, and be followed by incremental increases in complexity and responsibility/autonomy in practice. Tutor supervision needs to align with such gradual and incremental progression, being very direct initially and gradually moving to a model of pre‐ and de‐briefing. In our study, we have shown how this then enabled trainees to gradually, safely, and confidently take an increasingly independent (yet supported) approach to their clinical, patient‐facing (and eventually autonomous) practice. Regular contact and meaningful feedback by GP tutors along with both planned/formal and opportunistic/informal learning were found to be essential to support this progression (Haynes et al., 2002).

In this study, whilst tutors provided informal feedback to trainees, formative assessment tools were used minimally. Drawing on evidence from medical education, formative assessment tools promote active, learner‐centred learning, accompanied by feedback from supervisors, and are perceived as having a positive effect on practice (Gooding et al., 2016; Preston et al., 2020; Thistlethwaite, 2012). Furthermore, incorporating such assessment tools into a more structured training programme in future would allow for formal assessment of trainees’ competencies to undertake patient‐facing activities in GP. Clear requirements will also ensure the expectations are well defined for both trainees and their tutors, and that set standards ensure all trainees experience equal and equitable access to a high‐quality learning experience.

Similar to previous research (Christou et al., 2020; Gray, 2019), GP placements involved gradual progression, which started with an effective induction period to ease the transition into (and understanding of) GP. This study confirmed the importance of GP tutors being pharmacists, to role‐model and support, and bridge the understanding of a clinical pharmacist's scope of practice amongst the GP team (Christou et al., 2020; Gray, 2019). The reasons for trainees starting with observing non‐clinical staff need to be explained, so that trainees understand their relevance.

A joined‐up approach is important, recognising that the GP placement is part of 12 months’ pre‐registration training, with GPhC standards/competences needing to be achieved over the total duration. Each partner in the base–GP pairing needs to recognise the transferability of skills developed rather than being concerned about trainees spending less time in any one sector. To facilitate such a joined‐up approach, good and regular communication and handover between the base and GP tutors and indeed a co‐ordinated approach to supervision are important. The importance of effective engagement and support between both tutors as a catalyst for better trainee integration within GP teams has been highlighted in previous evaluations (Christou et al., 2020; NHS Health Education England, 2016).

Previous studies suggest that 26‐week GP placements are beneficial in developing the pre‐registration trainees’ clinical knowledge and confidence (NHS Health Education England, 2016), whereas 4–8‐week GP placements limit trainees’ opportunities to conduct supervised patient‐facing activities (Christou et al., 2020). Evidence from medical education suggests that placements longer than 8 weeks enable learners to better integrate into multidisciplinary teams, develop more autonomy, and undertake more complex tasks (Thistlethwaite et al., 2013). Our findings suggest that 13 weeks in GP is an appropriate minimum duration, whilst 26 weeks provided more opportunities for potentially more complex clinical and consultation skills learning. A longer duration was considered particularly welcome for community pharmacy.

By purposively sampling study sites on the basis of key situational variables, our study demonstrated that all models of placement structure (block/split week) supported trainees’ learning and development. This was because flexibility in set‐up allowed pairing to establish what was most suitable for their local situation, and it enabled placement sites to create a learning environment that was learner‐centred. There did, however, appear to be a preference for block placements in hospital pairings, in effect turning a GP placement into one rotation. Split days/ weeks appeared to be favoured in community pharmacy pairings, particularly if pharmacy and GP were located in relatively close proximity.

One of the main benefits of GP placements appeared to be trainees’ development of consultation and clinical assessments skills, which appear more difficult to achieve in both hospital and community pharmacy settings (Bullen et al., 2019; Jee et al., 2017). The GPhC introduced changes to the initial education and training of pharmacists in January 2021 which include replacing the pre‐registration year with a foundation training year, which will follow a revised 4‐year MPharm programme. The intention is to ensure pharmacists are equipped for their future roles, with revised learning outcomes ensuring pharmacists gain the skills, knowledge, and attributes needed for pharmacists to be independent prescriber ready at the point of registration. In light of these new GPhC standards and their focus on clinical and patient‐centred skills (General Pharmaceutical Council., 2021), GP placements will be critical to support the development of these competences and capabilities.

Clarity on which pre‐registration trainee competencies should be achieved during GP placements is also important. Recognising further previous research indicates that pre‐registration learning in community pharmacy and hospital settings differs (Bullen et al., 2019; Jee et al., 2016, 2019). We suggest a broader governance framework with minimum expectations to ensure consistency across the whole 12 months of foundation, with the need for standardised processes across different placements also recognised internationally (Lucas et al., 2018). Policy makers may also consider placements in all three sectors (hospital, community pharmacy, and GP).

### 4.1 Strength and limitations

Using a dyad/triad sampling approach enabled data triangulation and generated a multi‐faceted understanding of factors impacting implementation of cross‐sector GP placements. To the authors’ knowledge, this is the first national evaluation of cross‐sector pre‐registration pharmacists in GP placements in England. Findings from this study are of great interest and importance currently due to the changes in primary care service provision that have taken place, the resultant greater opportunities for pharmacists in primary care, and the upcoming changes to undergraduate and foundation education and training.

A key limitation was that there was potential self‐selection bias, and findings may be more positive. Furthermore, this qualitative study only represents the views of those who participated, and findings may be somewhat limited in their generalisability. It is also important to acknowledge that other countries will have differences in models of primary care service delivery and training of pharmacists. Therefore, further research is needed to determine the feasibility of implementing our cross‐sector training model within different countries and contexts.

## 5 CONCLUSION

This study evaluated the implementation of cross‐sector pre‐registration placements in GP, and identified barriers to, and enablers of, ‘successful’ implementation. Key attributes of a successful pre‐registration cross‐sector training experience were identified and framed according to an implementation model which can inform policy reforms, including the new GPhC standards for the initial education and training of pharmacists and their focus on clinical and patient‐centred skills. After first piloting our implementation model through a feasibility study, it could also be applied by countries with similar advancements in pharmacy education and training.

## ETHICS APPROVAL AND CONSENT TO PARTICIPATE

This study received ethics approval by The University of Manchester Research Ethics Committee (Ref no. 2020–7914–16794) and NHS Health Research Authority (Ref no. NHS001659). Written or verbal consent to participate in the study was obtained from each participant prior to starting data collection.

## CONFLICT OF INTEREST

None.

## Supporting information

Supplementary Material

Supplementary Material

Appendix S3

## Data Availability

The data that support the findings of this study are available on request from the corresponding author. The data are not publicly available due to privacy or ethical restrictions.

## ENDNOTE

^1^
In most cases, the pre‐registration year follows completion of an MPharm degree, but there are some exceptions with intercalated or integrated 5‐year programmes.
