# Supplementary material for: Cross‐sector pre‐registration trainee pharmacist placements in general practice across England: A qualitative study exploring the views of pre‐registration trainees and education supervisors
Source: Health Soc Care Community. 2022 Mar 15;30(6):2330–40. doi: 10.1111/hsc.13783 (PMC10078633; doi:10.1111/hsc.13783)
Supplement: Supplementary file 1 — Supplementary Material [file HSC-30-2330-s002.docx]

# Supplementary file 1: GP placement objectives and outcomes provided by HEE

GP placement objectives set by HEE

| **GP placement objectives set by HEE** |
| --- |
| - Understand the role of the clinical pharmacist in general practice as well as the systems and processes that apply in general practice - Develop communication and consultation skills to interact effectively with patients and their carers, healthcare professionals and the public - Respond to medication queries, complete medicines reconciliation and undertake medication reviews - Conduct consultations with patients with a range of acute or chronic healthcare needs - Use a range of basic clinical assessment skills, including the rationale for undertaking each and how to interpret and act on the results - Interpret clinical data (medical notes entries, laboratory and other tests) - Monitor ongoing treatment and make recommendations for common chronic conditions - Understand the burden of multimorbidity and polypharmacy and the need to take an individualised and holistic approach to shared decision making - Promote health and make every contact count |

Expected outcomes of the gp placement

| Trainees undertaking **13** weeks in GP | Trainees undertaking **26** weeks in GP |
| --- | --- |
| *At the end of the placement in general practice the trainee should:*   - Have a good understanding of general practice and the role of the pharmacist as part of the team - Have undertaken a minimum of two assessed basic face to face patient medication reviews and received feedback.* - Undertake the basic clinical assessments listed in **Table 3** | *At the end of the placement in general practice the trainee should:*   - Have an in-depth understanding of general practice and the role of the pharmacist as part of the team - Undertaken a minimum of four assessed face to face patient medication reviews, at least one of which should be a more complex review and received feedback.** - Undertake the basic clinical assessments listed in **Table 3** - Have completed a quality improvement project/audit cycle |

**Examples of a basic medication review may include a review focused on one condition (e.g. asthma, hypertension), or a medication switch requiring a face to face consultation*

***Examples of more complex reviews include the review of multiple medications for more than one condition, patients with known adherence issues, or patients with communication issues*
