# Supplementary material for: Cross‐sector pre‐registration trainee pharmacist placements in general practice across England: A qualitative study exploring the views of pre‐registration trainees and education supervisors
Source: Health Soc Care Community. 2022 Mar 15;30(6):2330–40. doi: 10.1111/hsc.13783 (PMC10078633; doi:10.1111/hsc.13783)
Supplement: Supplementary file 2 — Supplementary Material [file HSC-30-2330-s001.docx]

# Supplementary file 2: List of formative assessment tools

| **List of formative assessment tools** | | | | |
| --- | --- | --- | --- | --- |
| **Evidence Attribute** | **Mini-Clinical Evaluation Exercise**  **(Mini-CEX)** | **Intervention Recording**  **(IR)** | **Case Based Discussion**  **(CBD)** | **Medication Review and Consultation Assessment**  **(MRCA)** |
| **What does the tool support the development of?** | Behaviour  Judgement and reasoning in a range of clinical scenarios | Intervention  Recommending, justifying and communicating interventions | In-depth discussion  Depth and breadth of knowledge on a clinical area inspired by the management of a patient | Ability to deliver a medication review consultation  Ability to initiate, participate and conclude a patient-centred medication review consultation |
| **When to use?** | Real-time | Retrospectively | Retrospectively | Real-time |
| **Preparation required?** | No | Yes – up to 45 mins | Yes – 2 hours | Yes – up to 1 hour (data gathering) |
| **Time taken** | 5-15 mins | 15 mins | 30-40 mins | 20-30 mins |
| **Who can complete tool with pre-reg?** | Any healthcare professional  with knowledge in clinical area | Any healthcare professional  with knowledge in clinical area | Any healthcare professional  with knowledge in clinical area | Any healthcare professional  with knowledge in clinical area |
| **Example of when tool could be used** | Discussion of clinical reasoning decisions in real-life scenarios  • Responding to a medication query  • Conducting medicines reconciliation  • Medication Review | When a clinical intervention is being considered by the trainee  • Medicines reconciliation - intervention picked up as a result  • Responding to a medication query  • Medication review  • Chronic disease review | To explore a complex patient and their care in greater depth in order to deepen understanding of disease/medicine  • Complex medication regime reduced (deprescribing)  • Complex medicines reconciliation process  • Complex medical condition with specific medication regime | When the trainee delivers a supervised face to face medication related consultation for a patient |
| **Other information** | More exercises completed for range of activities, better it is for informing further development | Snapshot recording of interventions made to improve patient care | Discussion that can be presented as a case study on a chosen patient to demonstrate learning and development | Summary assessment of a range of activities |

**With the exception of the medication review and consultation assessment (MRCA), there was no expectation of when and how the other assessment tools were to be used.*
