# Supplementary material for: Cross‐sector pre‐registration trainee pharmacist placements in general practice across England: A qualitative study exploring the views of pre‐registration trainees and education supervisors
Source: Health Soc Care Community. 2022 Mar 15;30(6):2330–40. doi: 10.1111/hsc.13783 (PMC10078633; doi:10.1111/hsc.13783)
Supplement: Supplementary file 3 — Appendix S3 [file HSC-30-2330-s003.docx]

Appendix- S3

Pre-registration Pharmacist in General Practice – Trainee Interviews

| This topic guide provides the key themes and sub-themes to be explored in interviews with pre-registration pharmacists (trainees) who have enrolled in the pre-registration pharmacist in general practice project. It is not a set script.  The phrasing, pacing and ordering of questions should be tailored to reflect the individual respondent and the flow of the discussion in each interview.  Interviews are expected to last 30-40 minutes. |
| --- |

Motivations for undertaking cross-sector placements

- Why did you choose to take part in this cross-sector placement?

***Probe****: was it your first choice?*

What were you initially hoping/expecting you might gain?

Why did you choose this particular placement site?

- ***Prompts:***
  - *Location*
  - *Word-of-mouth*
  - *MPharm experience*

The structure of the training year

- Could you describe the structure/set-up for spending time between your employing sector and the general practice? I.e. how many days a week, for how many weeks? [Confirm placement length]
- What do you think about this structure/set-up?
- ***Prompts:*** *Would you prefer blocks; split weeks/days; a mix of both? Why?*
- How well do you feel you are/were able to manage the transition between your employing sector and the general practice?
- ***Probes:*** *Were there any particular challenges? On the other hand, was there anything that made the transition easier?*
- What do you think about the timing of your general practice placement in relation to your overall pre-registration year?
- What do you think about the duration of your general practice placement?
- ***Probe****: do you think it is sufficient?* ***If no:*** *would you prefer more/less time?*

How would you describe your work in the general practice?

- Probe: Is/was there a daily routine or was it different every time?

The knowledge and skills gained by trainees

Could you describe the activities you have undertaken in general practice?

- ***Prompts:***
  - *Patient medication reviews*
  - *Responding to medication queries*
  - *Medicines reconciliation*
  - *Basic clinical assessments*
  - *Interpreting clinical data*
  - *Shadowing general practice staff*
  - *Attending staff meetings*
  - *patient contact (on the phone and in person)*
- ***Probe:*** *do you feel you had adequate opportunities to undertake these activities?*
- How confident did you feel in carrying out these types of activities when you first stated?
- ***Probe:*** *How closely do they need to be supervised while doing them? Does this change over time?*  *Difference between when you first started until now/the end?*

Are there any skills that you have learnt/developed in general practice?

- ***Prompts:***
  - *Communication/consultation skills*
  - *Clinical skills*
  - *Leadership skills*
  - *Being able to work (more closely) with other healthcare professionals*
- ***Probe:*** *are there any skills that you have learnt/developed* over and above those learnt in other sectors?
- *How much do you feel your skills/knowledge have expanded/improved since starting your general practice placement?*
- *Is there anything you expected to learn/do that you haven’t been able to?*
- As time goes on, how useful do you feel you become in the practice?
- Do you feel you now have a good understanding of the role of the pharmacist as part of the general practice team?
- ***Probe:*** *What about the roles of other members of the general practice team?*
- Are/were there any areas that you would have liked to have seen covered during your general practice placement that are/were not?

Supervision models

- How well did you feel your employing sector pharmacist tutor helped you to prepare for your general practice placement?
- How well did you feel the general practice pharmacist tutor helped you to prepare for your general practice placement?
- ***Probe:*** *did you find the induction to be helpful?*
- How prepared did you find the staff at the general practice for your arrival?
- How did your general practice pharmacist tutor contribute to your learning?
- ***Probe:*** *do you feel this approach/method of learning was effective?*
- ***Prompts:*** *did they use any of the formative assessment tools? Are/were they helpful?*
- *Can you give me examples of how they used these assessment tools?*
- Do/did you find your general practice pharmacist tutor to be supportive?
- ***Probe:*** *How supportive do/did you find them? Can you give me some examples of how your tutor has supported your professional development?*
- Were there any ways in which you felt your general practice pharmacist tutor could have been more supportive?
- ***Probe:*** *Could you give examples in what ways you felt they were less supportive?*
- What do you think about the availability of your general practice pharmacist tutor?
- Do/did you find your employing sector pharmacist tutor to be supportive whilst on the general practice placement?
- ***Probe:*** *How supportive do/did you find them? Can you give me some examples of how they have supported you?*
- Were there any ways in which you felt your employing sector pharmacist tutor could have been more supportive?
- ***Probe:*** *Could you give examples in what ways you felt they were they less supportive?*
- Could you describe how you communicate/interact with your tutors at both sites?
- ***Probes:*** *how frequently do/did you communicate with them:*
  - *Whilst on the GP placement?*
  - *Whilst on the main employing sector?*
- *Are/were the both of them involved in assessing your training?*
- *Do you think your communication with both of your tutors could be improved?*
- What do you think about the communication/interaction between both of your tutors?
- ***Probe:*** *Do you think the communication between your tutors could be improved?*
- *How about appraisals? how they were conducted shared between the tutors*

The role of interprofessional and multidisciplinary learning

- Did you have the opportunity to engage with other healthcare professionals? Could you describe this?
- ***Probe:*** *How do you feel they contributed to your learning experience?*
- Did you find other healthcare professionals to be supportive?
- ***Probe:*** *Which ones? Could you give an example of ways in which they were supportive?*
- Did you have the opportunity to engage with any other staff in general practice? Could you describe this?
- ***Probe:*** *How do you feel they contributed to your learning experience?*
- Did you find other staff in the general practice to be supportive?
- ***Probe:*** *Which ones? Could you give an example of ways in which they were supportive?*
- Were you the only trainee in the general practice site?
- ***Probe:*** *do you think that having another trainee on site changed/ would have changed your learning experience?*
- Was there any peer support available to you? Could you describe this?
- ***Probe:*** *How did this help?* ***If not:*** *what would have helped you?*
- Did you have the opportunity to engage with trainees from other professions? Could you describe this?

The (educational) support

- Do you feel there was sufficient working space during your training in the general practice site? What about access to electronic hardware?
- Did you find any of the resources provided for your pre-registration training to be helpful?
- ***Prompts:***
  - *HEE resources( e.g. handbook, development/quality framework, suggested activities, TRAS guidance, resources/formative assessments, E-portfolio)*
  - *GPhC resources*
  - *Online resources*
  - *CPPE learning programmes*
- ***Probe:*** *is there anything else that you feel (would have) helped prepare you for your placement?*
- How did you monitor your progress throughout the placement?
- ***Prompt:*** *did you use the E-portfolio? Was it helpful?*
- ***Probe:*** *Is there anything else that you feel could be done to improve the monitoring of your training?*

The cost and gain

- What do you think have been the main benefits/value of doing a split placement?
- ***Probe****: Do you feel that there have been any positive effects on the general practice team?*
- *Do you feel that there have been any positive effects upon returning to the main employing sector?* **(If they returned to the base sector)**
- On the other hand, do you think there have been any disadvantages/negative effects of doing a split placement?
- ***Probe****: Do you feel that there have been any negative effects on the general practice team?*
- *What about spending time away from your main employing sector?*
- From your knowledge, how would you compare your learning/development to other pre-registration trainees in single-sector placements?

Final reflections

- Has this training experience changed the way you think of yourself as a pharmacist?
- If you were giving advice to someone else who was doing this type of placement, what would it be?
- Would you consider general practice pharmacy as a future career option?
- ***Probe****: Has this changed at all?*

Close

- Thank you for sharing your views and experiences. Is there anything else you would like to add?

Pre-registration Pharmacist in General Practice –Supervisor in Employing Pharmacy Interviews

| This topic guide provides the key themes and sub-themes to be explored in interviews with educational supervisors (tutors) in the employing pharmacy who have enrolled in the pre-registration pharmacist in general practice project. It is not a set script.  The phrasing, pacing and ordering of questions should be tailored to reflect the individual respondent and the flow of the discussion in each interview.  Interviews are expected to last 30-40minutes. |
| --- |

Motivations for undertaking cross-sector placements

- Why did you choose to take part in this cross-sector placement?
- How many years now have you been running this placement?

What were you initially hoping/expecting you might gain?

- - ***Prompts:*** *Benefits for self? Benefits for patients? Benefits for pharmacy profession?*

Did you find difficulties/challenges setting up the cross-sector placement?

The structure of the training year

- Could you describe the structure/set-up for how the trainee spends time between their employing sector and the general practice? I.e. how many days a week, for how many weeks? [Confirm placement length]
- What do you think about this structure/set-up?
- ***Prompts:*** *Would you prefer blocks; split weeks/days; a mix of both? Why?*
- How well do you feel the trainee was able to manage the transition between their employing sector and the general practice?
- ***Probes:*** *Were there any particular challenges? On the other hand, was there anything that made the transition easier?*
- What do you think about the timing of the general placement in relation to the overall pre-registration year?
- What do you think about the duration of the general practice placement?
- ***Probe****: do you think it is sufficient?* ***If no:*** *would you prefer more/less time?*

The knowledge and skills gained by trainees

**Note: Some of these questions will be difficult to answer depending on the model (i.e. block placement) unless the trainee has returned to the pharmacy sites.**

Are you aware of any activities the trainee has undertaken in general practice?

- ***Prompts:***
  - *Patient medication reviews*
  - *Responding to medication queries*
  - *Medicines reconciliation*
  - *Basic clinical assessments*
  - *Interpreting clinical data*
  - *Shadowing general practice staff*
  - *Attending staff meetings*
- ***Probe:*** *do you feel they had adequate opportunities to undertake these activities?*
- How confident do/did you feel in the trainee’s ability to carry out these types of activities?
- Are there any skills that your trainee has learnt/developed since starting their training in general practice?
- ***Prompts:***
  - *Communication/consultation skills*
  - *Clinical skills*
  - *Leadership skills*
  - *Being able to work (more closely) with other healthcare professionals*
- ***Probe:*** *are there any skills that they have learnt/developed* over and above those learnt in [interviewee’s sector]?
- *How much do you feel their skills/knowledge have expanded/improved since starting the general practice placement?*
- Have they been able to apply these skills in the pharmacy sector?
- Have you seen any difference their ability (confidence, competence etc.)
- Are/were there any areas that you would have liked to have seen covered during the trainee’s general practice placement that are/were not?

Supervision models

- How well did you feel you prepared the trainee for their general practice placement?
- ***Probe:*** *did you find the induction to be helpful?*
- How do/did you support your trainee during the general practice placement?
- ***Probe:*** *Can you give me some examples of how you have supported their professional development?*
- Were there any areas where you felt you could have been more supportive?
- ***Probe:*** *Could you give examples in what ways you felt you were less supportive?*
- How did you monitor the trainee’s progress in the general practice placement?
- ***Prompt:*** *did you use the E-portfolio? Was it helpful?*
- ***Probe:*** *Is there anything else that you feel could be done to improve the monitoring of the training?*
- Do/did you find the general practice pharmacist tutor to be supportive of the trainee?
- ***Probe:*** *How supportive do/did you find them? Can you give me some examples of how they supported the trainee?*
- Were there any areas you felt the general practice pharmacist tutor could have been more supportive?
- ***Probe:*** *Could you give examples in what ways you felt they were they less supportive?*
- Could you describe how you communicate and interact with the tutor at the other site?
- ***Probes:*** *how frequently do/did you communicate with each other?*
- *Are/were the both of you involved in the assessment and appraisal of the trainee?*
- *Do you think this communication process could be improved?*

The (educational) support

- Was your trainee the only trainee in the general practice site?
- ***Probe:*** *do you think that having another trainee on site changed/ would have changed their learning experience?*
- Was there any peer support available to your trainee? Could you describe this?
- ***Probe:*** *How did this help them?* ***If not:*** *what would have helped them?*
- Did you find any of the resources provided to supervise the trainee during the split placement to be helpful?
- ***Prompts:***
  - *HEE resources( e.g. handbook, development/quality framework, E-portfolio)*
  - *GPhC resources*
  - *Online resources*
  - *CPPE learning programmes*
- ***Probe:*** *is there anything else that you feel (would have) helped prepare you for your supervision role in split placements?*
- What about the support provided by HEE i.e. the regional facilitators – were they helpful?

–  ***Probe:*** Availability and responsiveness? *Could they have done more? Should they have been less involved?*

- Do you think there were clear and appropriate indemnity arrangements in place for the trainee, their tutors and employers?

The cost and gain

- What do you think have been the main benefits/value of your trainee doing a split placement?
- ***Probe****: Do you feel that there have been any positive effects upon returning to the main employing sector?*
- On the other hand, do you think there have been any disadvantages/negative effects of your trainee doing a split placement?
- ***Probe****: What about the trainee spending time away from their main employing sector?*
- From your knowledge, how would you compare your trainee’s learning/development to other pre-registration trainees in single-sector placements?
- ***Probe****: Personal Impact: has it been more work for them? Have they built additional relationships?*

Final reflections

- What do you think made this pre-reg training experience successful/unsuccessful?
- What would you say has been the best aspect of this innovation for you?
- What has been the biggest challenge?
- Would you consider supervising a pre-reg pharmacist doing a split placement again in the future?

Close

- Thank you for sharing your views and experiences. Is there anything else you would like to add?

Pre-registration Pharmacist in General Practice –Supervisor in General Practice Interviews

| This topic guide provides the key themes and sub-themes to be explored in interviews with educational supervisors (tutors) in general practice who have enrolled in the pre-registration pharmacist in general practice project. It is not a set script.  The phrasing, pacing and ordering of questions should be tailored to reflect the individual respondent and the flow of the discussion in each interview.  Interviews are expected to last 30-40 minutes. |
| --- |

Motivations for undertaking cross-sector placements

- Why did you choose to take part in this cross-sector placement?
- How many years now have you been running this placement?

What were you initially hoping/expecting you might gain?

- - ***Prompts:*** *Benefits for self? Benefits for patients? Benefits for employer? Benefits for pharmacy profession?*

Did you find difficulties/challenges setting up the cross-sector placement?

The structure of the training year

- Could you describe the structure/set-up for how the trainee spends time between their employing sector and the general practice? I.e. how many days a week, for how many weeks? [Confirm placement length]
- What do you think about this structure/set-up?
- ***Prompts:*** *Would you prefer blocks; split weeks/days; a mix of both? Why?*
- How well do you feel the trainee was able to manage the transition between their employing sector and the general practice?
- ***Probes:*** *Were there any particular challenges? On the other hand, was there anything that made the transition easier?*
- What do you think about the timing of the general practice placement in relation to the overall pre-registration year?
- What do you think about the duration of the general practice placement?
- ***Probe****: do you think it is sufficient? If no: would you prefer more/less time?*
- Tell me about the practice in which you work, what is your working pattern?
- ***Probe:*** *How can a pre-registration pharmacist assist in this work?*

The knowledge and skills gained by trainees

Could you describe the activities the trainee has undertaken in general practice?

- ***Prompts:***
  - *Patient medication reviews*
  - *Responding to medication queries*
  - *Medicines reconciliation*
  - *Basic clinical assessments*
  - *Interpreting clinical data*
  - *Shadowing general practice staff*
  - *Attending staff meetings*
  - *Patient facing activity*
- ***Probe:*** *do you feel they had adequate opportunities to undertake these activities?*
- How confident do/did you feel in the trainee’s ability to carry out these types of activities when they first started?
- ***Probe:*** *How closely do they need to be supervised while doing them? Does this change over time? Difference between when they first started until now/the end?*
- Are there any skills that your trainee has learnt/developed since starting their training in general practice?
- ***Prompts:***
  - *Communication/consultation skills*
  - *Clinical skills*
  - *Leadership skills*
  - *Being able to work (more closely) with other healthcare professionals*
- ***Probe:*** *are there any skills that they have learnt/developed* over and above those learnt in other sectors?
- *How much do you feel their skills/knowledge have expanded/improved since starting the general practice placement?*
- As time goes on, how useful do you feel they become to the general practice team?
- Do you feel your trainee now has a good understanding of the role of the pharmacist as part of the general practice team?
- ***Probe:*** *What about the roles of other members of the general practice team?*
- Are/were there any areas that you would have liked to have seen covered during the trainee’s general practice placement that are/were not?

Supervision models

- How prepared was the trainee for their general practice placement?
- ***Probe:*** *did you find the induction to be helpful?*
- How prepared were the staff at the general practice for the arrival of the trainee?
- How did you contribute to the trainees learning and development during the placement?
- ***Probe:*** *Do you feel this approach/method of learning was effective?*
- How did you structure the trainees’ timetable?
- ***Prompts:*** *did you use any of the formative assessment tools {Mini-CEX, Intervention recording, Case-based discussion, MRCA}? Are/were they helpful?*
- *Can you give me some examples of how you used these assessment tools?*
- *What about other ways of learning?*
- How do/did you support your trainee during the general practice placement?
- ***Probe:*** *Can you give me some examples of how you have supported their professional development?*
- Were there any areas where you felt you could have been more supportive?
- ***Probe:*** *Could you give examples in what ways you felt you were less supportive?*
- What do you think about your availability for supervising the trainee?
- ***Probe:*** *What can your trainee do when you are not there?*
- How did you monitor the trainee’s progress throughout the placement?
- ***Prompt:*** *did you use the E-portfolio {VQmanager}? Was it helpful?*
- ***Probe:*** *Is there anything else that you feel could be done to improve the monitoring of the training?*
- Do/did you find the employing sector pharmacist tutor to be supportive of the trainee whilst on the general practice placement?
- ***Probe:*** *How supportive do/did you find them? Can you give me some examples of how they supported the trainee?*
- Were there any areas you felt the employing sector pharmacist tutor could have been more supportive?
- ***Probe:*** *Could you give examples in what ways you felt they were they less supportive?*
- Could you describe how you communicate and interact with the tutor at the other site?
- ***Probes:*** *how frequently do/did you communicate with each other?*
- *Are/were the both of you involved in the assessment and appraisal of the trainee?*
- *Do you think this communication process could be improved?*

The role of interprofessional and multidisciplinary learning

- Did your trainee have the opportunity to engage with other healthcare professionals? Could you describe this?
- ***Probe:*** *How do you feel they contributed to their learning experience?*
- Did you find other healthcare professionals to be supportive of the trainee?
- ***Probe:*** *Which ones? Any ways in which they were supportive?*
- Did your trainee have the opportunity to engage with any other staff in general practice? Could you describe this?
- ***Probe:*** *How do you feel they contributed to their learning experience?*
- Did you find other staff in the general practice to be supportive of the trainee?
- ***Probe:*** *Which ones? Any ways in which they were supportive?*
- Was your trainee the **only pharmacist trainee** in the general practice site?
- ***Probe:*** *do you think that having another trainee on site changed/ would have changed their learning experience?*
- Did they have the opportunity to engage **with trainees from other professions**? Could you describe this?

The (educational) support

- Do you feel there was sufficient working space for the trainee during their training in the general practice site? What about access to electronic hardware?
- Did you find any of the resources provided to supervise the trainee to be helpful?
- ***Prompts:***
  - *HEE resources( e.g. handbook, development/quality framework, suggested activities, TRAS guidance, resources/formative assessments, E-portfolio)*
  - *GPhC resources*
  - *Online resources*
  - *CPPE learning programmes*
- ***Probe:*** *is there anything else that you feel (would have) helped prepare you for your supervision role?*
- What about the support provided by HEE i.e. the regional facilitators – were they helpful?

–  ***Probe:*** Availability and responsiveness? *Could they have done more? Should they have been less involved?*

- What was the impact on you as an individual
- ***Probe:*** *additional time pressures vs ability of the trainee to support work? Was it a rewarding experience?*

The cost and gain

- What do you think have been the main benefits/value of your trainee doing a split placement?
- ***Probe****: Do you feel that there have been any positive effects on the general practice team?*
- On the other hand, do you think there have been any disadvantages/negative effects of your trainee doing a split placement?
- ***Probe****: Do you feel that there have been any negative effects on the general practice team?*
- From your knowledge, how would you compare your trainee’s learning/development to other pre-registration trainees in single-sector placements?

Final reflections

- What do you think made this pre-reg training experience successful/unsuccessful?
- What has been the biggest challenge?
- Would you consider supervising a pre-reg pharmacist in general practice again in the future?

Close

- Thank you for sharing your views and experiences. Is there anything else you would like to add?
